# Iontosomes: Electroresponsive Liposomes for Topical Iontophoretic Delivery of Chemotherapeutics to the Buccal Mucosa

**DOI:** 10.3390/pharmaceutics13010088

**Published:** 2021-01-11

**Authors:** Kiran Sonaje, Vasundhara Tyagi, Yong Chen, Yogeshvar N. Kalia

**Affiliations:** 1 School of Pharmaceutical Sciences, University of Geneva, CMU-1 Rue Michel Servet, 1211 Geneva, Switzerland; kiransonje@gmail.com (K.S.); vasundhara.tyagi412@gmail.com (V.T.); scuchen2003@ntu.edu.cn (Y.C.); 2 Institute of Pharmaceutical Sciences of Western Switzerland, University of Geneva, CMU-1 Rue Michel Servet, 1211 Geneva, Switzerland

**Keywords:** iontophoresis, deformable liposomes, nanocarrier, oral cancer, drug delivery, buccal mucosa

## Abstract

The targeted local delivery of anticancer therapeutics offers an alternative to systemic chemotherapy for oral cancers not amenable to surgical excision. However, epithelial barrier function can pose a challenge to their passive topical delivery. The charged, deformable liposomes—“iontosomes”—described here are able to overcome the buccal mucosal barrier via a combination of the electrical potential gradient imposed by iontophoresis and their shape-deforming characteristics. Two chemotherapeutic agents with very different physicochemical properties, cisplatin (CDDP) and docetaxel (DTX), were co-encapsulated in cationic iontosomes comprising 1,2-dioleoyl-3-trimethylammonium-propane (DOTAP) and Lipoid-S75. The entrapment of CDDP was improved by formulating it in anionic reverse micelles of dipalmitoyl-sn-glycero-3-phospho-rac-glycerol sodium (DPPG) prior to loading in the iontosomes. Cryo-TEM imaging clearly demonstrated the iontosomes’ electroresponsive shape-deformable properties. The in vitro transport study using porcine mucosa indicated that iontosomes did not enter the mucosa without an external driving force. However, anodal iontophoresis resulted in significant amounts of co-encapsulated CDDP and DTX being deposited in the buccal mucosa; e.g., after current application for 10 min, the deposition of CDDP and DTX was 13.54 ± 1.78 and 10.75 ± 1.75 μg/cm^2^ cf. 0.20 ± 0.07 and 0.19 ± 0.09 μg/cm^2^ for the passive controls—i.e., 67.7- and 56.6-fold increases—without any noticeable increase in their transmucosal permeation. Confocal microscopy confirmed that the iontosomes penetrated the mucosa through the intercellular spaces and that the penetration depth could be controlled by varying the duration of current application. Overall, the results suggest that the combination of topical iontophoresis with a suitable nanocarrier system can be used to deliver multiple “physicochemically incompatible” chemotherapeutics selectively to oral cancers while decreasing the extent of systemic absorption and the associated risk of side effects.

## 1. Introduction

A major challenge in treating any cancer is achieving a high cure rate while preserving vital functions of the affected tissue. This is especially important for oral cancers where the affected regions are essential for regular human activities [1]. Although the surgical resection of such cancers can permanently impair normal functioning, the outcome of radiotherapy alone can be unsatisfactory, particularly for advanced stage cancer. Therefore, systemic chemotherapy has been increasingly incorporated into treatment protocols in recent years [1,2]. However, the incidence of off-site adverse events induced by systemic chemotherapy can compromise the overall success of such treatments. Given this scenario, a topical delivery system enabling the local administration of chemotherapeutic agents for oral cancer treatment would offer a non-invasive, targeted and patient-friendly alternative to their systemic, frequently intravenous, administration. However, the oral mucosa represents a significant barrier to the transport of drugs with unfavorable physicochemical properties [3]. Another challenge in topical buccal delivery is the need to achieve therapeutically significant drug levels in the affected tissue quickly, since only short duration applications are practical in the oral cavity due to obvious anatomical/functional constraints [4].

Iontophoresis, a non-invasive technique involving the application of a mild electric current to enhance the penetration of water-soluble, ionizable drugs, offers a simple, effective, and controlled method to deliver chemotherapeutics rapidly into the buccal mucosa [5]. The electric potential acts as a second driving force in addition to the concentration gradient, and this results in increased drug delivery rates as compared to passive drug diffusion alone. The amount of drug delivered can be controlled by modulating the intensity and duration of current application, making personalized dosing feasible [6]. We have previously demonstrated the successful concurrent delivery of 5-fluorouracil (5-FU) and leucovorin (LV; folinic acid) to buccal mucosa using short duration iontophoresis for the treatment of head and neck cancers [5]. However, one drawback in delivering free-form chemotherapeutics using this technique is the possibility of their systemic clearance and the risk of exposure to associated side effects. Moreover, not all chemotherapeutic agents can be considered as suitable candidates for iontophoretic delivery; e.g., non-polar molecules with poor aqueous solubility and lacking ionizable functional groups are unsuited to this technique [7]. Therefore, a nanocarrier system capable of encapsulating chemotherapeutics with diverse physicochemical properties and enabling sustained post-iontophoretic release in the buccal mucosa after short duration iontophoresis would be of considerable interest.

The oral mucosa is composed of several layers of tightly packed epithelial cells that form the primary barrier to drug permeation [8,9,10]. The intercellular space in these layers is believed to be narrower than 20 nm [8]. Hence, traditional nanocarriers such as polymeric nanoparticles or conventional liposomes are unlikely to cross this barrier by passive diffusion. In recent years, various deformable vesicles such as Transferosomes^®^, niosomes, or ethosomes have been introduced for transdermal drug delivery [11,12,13,14]. Transferosomes^®^ and niosomes are composed of an edge activator, which is a surfactant that destabilizes the lipid bilayer and provides elasticity to the liposomes. In the case of ethosomes, ethanol imparts flexibility to vesicles via its interdigitation into the lipid bilayers. It has been assumed that the elastic nature of such deformable vesicles allows them to squeeze between the corneocytes and traverse the epidermis [15]. Although the mechanism that drives their penetration remains unclear, it has been hypothesized that Transferosomes^®^ utilize the transdermal osmotic gradient resulting from differences in the hydration levels of the outer stratum corneum and inner viable epidermis to spontaneously penetrate the skin. However, unlike skin, the buccal mucosa is uniformly hydrated due to the presence of saliva, and this mechanism is unlikely to work for buccal delivery.

**Figure 1:**
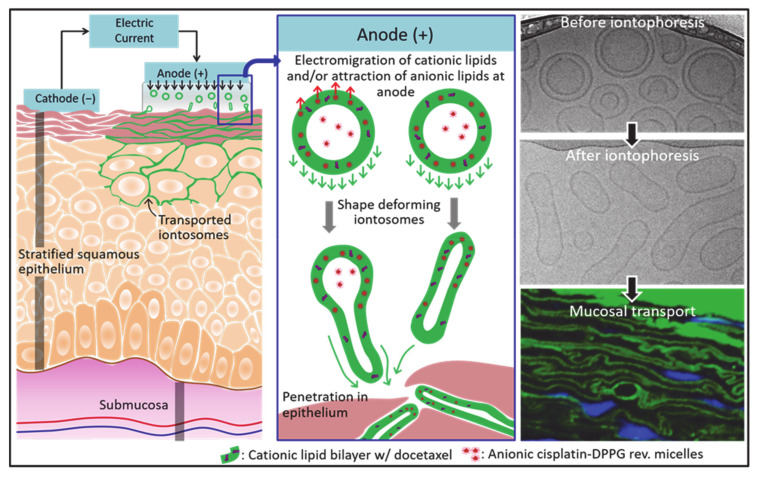
A schematic diagram showing the basic concept underlying anodal iontophoretic mucosal delivery of iontosomes employed and validated in this study. The cationic iontosomes (green) undergo shape deformation upon application of the current. During iontophoresis, the anionic reverse micelles (red) are attracted to the anode; at the same time, the cationic lipid bilayers electromigrate toward the mucosa. These opposing forces result in elongation of the iontosomes. Then, the deformed iontosomes enter the mucosa through the intercellular spaces in epithelium. The images on the right show the transmission electron microscopy at cryogenic temperature (cryo-TEM) and confocal micrographs of iontosomes during iontophoresis and after deposition into mucosa. The images on the right show the cryo-TEM and confocal micrographs from the current work (see below), showing the shape deformation of iontosomes after iontophoresis and upon deposition in the mucosa.

The present investigation describes the preparation and evaluation of novel cationic liposomes, which we have called “iontosomes”, for the effective iontophoretic delivery of chemotherapeutics across the buccal mucosa. Docetaxel (DTX) and cisplatin (CDDP), which are widely prescribed in combination for the treatment of head and neck cancers, were loaded in these carriers for simultaneous iontophoretic delivery. The iontosomes were engineered to undergo shape deformation in response to the applied electric field, which facilitates their penetration through the narrow intercellular spaces of the mucosal epithelium (Figure 1). They were formulated by combining a cationic lipid film with reverse micelles of an anionic lipid. The iontosomes were characterized in terms of theirsize, zeta potential, electro-responsive shape deformation, and in vitro drug release behavior. Their anti-tumoral activity was evaluated in vitro using HeLa cells. Finally, the mucosal iontophoretic delivery of these iontosomes was investigated in porcine esophageal mucosa, which is a validated model to study buccal permeation [16].

## 2. Materials and Methods

### 2.1. Materials

The different lipids used to formulate the iontosomes, 1,2-dioleoyl-3-trimethylammonium-propane chloride (DOTAP), soybean phosphatidylcholine (Lipoid-S75), and 1,2-Dipalmitoyl-sn-glycero-3-phospho-rac-glycerol sodium (DPPG) were generous gifts from Lipoid AG (Steinhausen, Switzerland). The chemotherapeutic agents, DTX and CDDP were obtained from Alfa Aesar GmbH (Karlsruhe, Germany) and Strem Chemicals, Inc. (Kehl, Germany), respectively. All other chemicals were of analytical reagent grade. The human cervical cancer cell line (HeLa) was obtained from American Type Culture Collection (ATCC, Rockville, MD, USA).

### 2.2. Formulation Development

#### 2.2.1. Preparation of Conventional Liposomes

Conventional cationic liposomes encapsulating DTX were prepared using the lipid film hydration method as described previously [17]. First, DOTAP (0.1 mmol), Lipoid-S75 (0.09 mmol), and DTX (0.01 mmol) were added to 15 mL of chloroform in a round-bottomed flask. The mixture was gently warmed to 40°C for 15 min, and the solvent was evaporated using a rotary evaporator (Rotavapor R-124, Buchi; Flawil, Germany) until a thin lipid film was formed. Solvent traces were removed by desiccating the film for 60 min at high vacuum. The lipid film was hydrated with an aqueous solution of CDDP (1 mg/mL, 10 mL) in 0.9% (*w*/*v*) NaCl. Large multilamellar liposomes were spontaneously formed upon addition of the CDDP solution. The lipid suspension was left overnight to allow swelling of the liposomes and the partitioning of CDDP into the liposomes. Then, the uniformly sized liposomes were obtained by extruding the initial suspension through a series of polycarbonate membrane filters of decreasing pore sizes (400, 200, and 100 nm, five cycles each) using a mini-extruder (Avanti Polar Lipids, Inc.; Alabaster, AL, USA) maintained at 40 °C. Finally, the formulated liposomes were purified using a Zeba™ spin desalting column (Life Technologies Europe B.V.; Zug, Switzerland) to remove the unentrapped drugs.

#### 2.2.2. Preparation of Iontosomes

**Figure 2:**
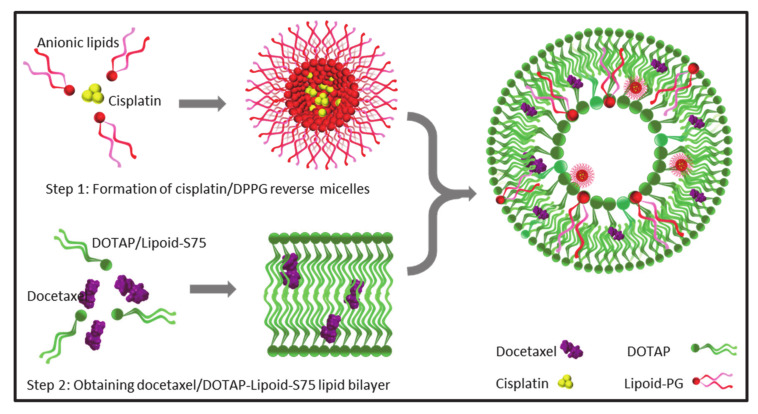
Formulation of electroresponsive iontosomes; cisplatin (CDDP) was formulated into the anionic reverse micelles using dipalmitoyl-sn-glycero-3-phospho-rac-glycerol sodium (DPPG) (red) and docetaxel (DTX) was loaded in the cationic lipid bilayer of 1,2-dioleoyl-3-trimethylammonium-propane soybean phosphatidylcholine (DOTAP-Lipoid-S75) (green). Then, the iontosomes were obtained by hydrating the cationic lipid bilayer with the CDDP-DPPG reverse micelles.

The iontosomes were obtained by mixing the aforementioned cationic lipid film with the CDDP-loaded reverse micelles of DPPG (Figure 2). The preparation of reverse micelles was based on Lipoplatin™, which is a successful liposomal formulation of cisplatin [18]. Briefly, DPPG (0.1 mmol) and CDDP (0.1 mmol) were added to 6 mL of Tris buffer (pH 7.5, 0.1 M) containing 30% ethanol (*v*/*v*), and the mixture was heated at 50 °C for 15−30 min. The heating step converted the suspended CDDP powder into a colloidal gel form. The reverse micelles were obtained by diluting the colloidal gel with 5% glucose solution (*w*/*v*) to a CDDP concentration of 2 mg/mL.

The formulated CDDP-DPPG reverse micelles were used to hydrate the DTX-loaded cationic lipid film described above. Three formulations at different concentration were prepared using increasing volumes of reverse micelles (2.5, 5.0, and 10 mL) (see below Table 1). After overnight hydration, the volume of the formulation was adjusted to 10 mL with glucose solution (5% *w*/*v*) where necessary, and large clumps of hydrated lipids were dispersed using a micropipette tip to obtain a homogeneous suspension of lipid vesicles. Then, the uniformly sized iontosomes were obtained by extrusion through polycarbonate membrane filters as described above.

#### 2.2.3. Preparation of Fluorescent Iontosomes

To prepare fluorescent iontosomes, Lipoid-S75 was labeled with an amine-reactive dye, NHS-fluorescein (Thermo Fisher Scientific, Reinach, Switzerland), as described previously [19]. Briefly, Lipoid-S75 (1 mmol) was reacted with NHS-fluorescein (0.1 mmol) in 10 mL of tetrahydrofuran containing 0.2 mL of triethylamine at room temperature for 4 h. The reaction mixture was dried under vacuum, and the product was dissolved in 100 mL of chloroform. The fluorescent lipid was purified to remove the unreacted dye by liquid–liquid extraction with MilliQ water until no fluorescence was observed in the aqueous phase. The purity of the fluorescein-labeled lipid was confirmed using reversed phase HPLC [20]. The fluorescent iontosomes were prepared as described above except that half of the Lipoid-S75 was substituted with its fluorescent derivative.

#### 2.2.4. Preparation of Solution Formulations

The solution formulations of CDDP and DTX were used as controls in the subsequent studies. Although CDDP is slightly soluble in water (solubility ≈2.5 mg/mL), it isomerizes into the inactive *trans*-form in chloride-free medium [21]. Therefore, a stock solution of CDDP at 1 mg/mL was prepared in normal saline (0.9% NaCl *w*/*v*). The availability of excess chloride ions prevents the isomerization of CDDP in saline and provides stable solutions that retain CDDP activity for at least 30 days [22,23].

The preparation of DTX solution was based on its commercial formulation, Taxotere^®^. Briefly, 20 mg of DTX was added to 0.5 mL of Tween 80 (520 mg) and vortexed until a clear solution was obtained. Then, the resulting DTX solution was mixed with 1.5 mL of 13% ethanol in PBS (pH 7.4), resulting in a 1% *w*/*v* stock solution of DTX. Both stock solutions were stored in the dark at 2–8°C for no longer than four weeks. These stock solutions were diluted to the required working concentrations for respective experiments using appropriate vehicles.

### 2.3. Characterization of Iontosomes

#### 2.3.1. Physicochemical Characteristics and Drug Content

The particle size and zeta potential of the formulated iontosomes were analyzed by dynamic light scattering (DLS) using a Zetasizer Nano-ZS (Malvern Instruments, Malvern, UK) at 25 °C. All measurements were made in triplicate.

To determine the drug content, purified liposomes or iontosomes were disrupted by 0.1% Triton X-100, and amounts of DTX and CDDP were quantified using the validated HPLC-UV method (Supplementary Materials). The encapsulation efficiency (EE) was calculated using the following equation:

(1) $$\mathrm{Encapsulation}\;\mathrm{efficiency}\left(\%\right)=\frac{\mathrm{Amount}\;\mathrm{of}\;\mathrm{drug}\;\mathrm{incorporated}\;\mathrm{in}\;\mathrm{iontosomes}\times 100}{\mathrm{Intial}\;\mathrm{mass}\;\mathrm{of}\;\mathrm{the}\;\mathrm{drug}}.$$

#### 2.3.2. Cryo-Transmission Electron Microscopy (Cryo-TEM)

The shape and morphology of the formulated reverse-micelles, liposomes, and iontosomes were studied using transmission electron microscopy at cryogenic temperature (Cryo-TEM) using a previously reported protocol [24]. Briefly, 5 μL of each formulation was applied to a copper grid pre-coated with perforated carbon film. After blotting the excess formulation, the grid was immediately immersed in liquid ethane container cooled using liquid nitrogen. Cryo-TEM images were recorded at −170 °C using a Philips CM12 transmission electron microscope (Eindhoven, The Netherlands) operating at 100 kV and equipped with a cryo-specimen holder Gatan 626 (Warrendale, PA, USA). Digital images were recorded with a Gatan MultiScan CCD camera and processed using the Gatan Digital Micrograph.

#### 2.3.3. Effects of Iontophoresis on Drug Release and Iontosome Characteristics

Drug release from the iontosomes was tested using a dialysis membrane with a 6–8 kDa molecular weight cut-off (Spectrapor^®^ 3, Spectrum Laboratories, New Brunswick, NJ, USA) mounted between the donor and receptor compartments of Franz diffusion cells. The donor cell was filled with 0.5 mL of formulation and the receptor cell contained 4.5 mL of 0.1% (*w*/*v*) Tween 80 in phosphate-buffered saline (PBS, pH 7.4). The anode was connected to the donor compartment via a salt bridge assembly (3% agarose with 100 mM NaCl). Constant current (0.5 mA/cm^2^) was applied using Ag/AgCl electrodes connected to a power supply (Kepco^®^ APH 1000 M; Flushing, NY, USA). The electric current was applied for 20 min followed by passive release in vitro for 12 h.

At predetermined intervals, aliquots (500 μL) of the receptor medium were withdrawn and immediately replaced with an equal volume of fresh buffer. In addition, the amounts of unreleased drug were determined in the residual formulation and dialysis membranes after the experiment. The amounts of CDDP and DTX in the samples were determined using the respective HPLC-UV analytical methods. Finally, the size and morphology of iontosomes after exposure to the iontophoretic conditions were determined using the zetasizer and Cryo-TEM, respectively.

#### 2.3.4. Evaluation of In Vitro Anti-Tumoral Activity of Iontosomes

A human cervical cancer cell line (HeLa cells, purchased from ATCC) was employed for the evaluation of the anti-tumoral activity of the formulated iontosomes in vitro. The cells were cultured in Dulbecco’s modified Eagle medium (DMEM, high glucose, GlutaMAX™) supplemented with 10% fetal calf serum, 100 U/mL penicillin, and 100 μg/mL streptomycin (all from Invitrogen Life Technologies; Basel, Switzerland) in a humidified atmosphere with 5% CO_2_ at 37 °C.

The cells were seeded in 96-well plates at a density of 104 cells (100 µL) per well and incubated for 24 h to allow cell attachment. After 24 h, the medium in the wells was replaced with DMEM containing drug-loaded iontosomes, solution formulations of CDDP and DTX, or their combination and incubated for 24 h. Drug concentrations in the solution formulations were equivalent to those in the iontosomes. Blank iontosomes were also tested to evaluate whether the lipids exhibited any cytotoxic activity on the HeLa cells. After 24 h treatment, DMEM containing MTT ((3-(4,5-Dimethylthiazol-2-yl)-2,5-Diphenyltetrazolium Bromide); 20 µL, 5 mg/mL) was added, and cells were incubated for an additional 4 h. Finally, the medium containing MTT was aspirated and formazan crystals formed by viable cells were dissolved by the addition of DMSO (100 µL). Absorbance was measured at 570 nm using a microplate reader (Synergy™ MX, BioTek Instruments; Luzern, Switzerland). Untreated cells were considered as controls representing 100% viability.

#### 2.3.5. Visualization of Cellular Uptake Studies Using Confocal Microscopy

To visualize the cellular uptake of iontosomes, the cells were seeded on glass coverslips in 12-well plates (105 cells/well). After incubating the cells for 24 h, the medium was replaced with fluorescent iontosomes and incubated for a further 4 h. After the treatment, cells were washed thrice with PBS and fixed using paraformaldehyde (PFA, 4% *w*/*v*) in PBS at room temperature for 15 min. The fixed cells were counterstained with Hoechst 33258, and the coverslips were mounted on glass slides with 60% glycerol as mounting medium. Cells were scanned, and images were recorded with a CLSM microscope (Zeiss LSM700, Carl Zeiss Microscopy GmbH, Jena, Germany).

### 2.4. Mucosal Transport Studies

#### 2.4.1. Mucosa Source

The mucosal transport of iontosomes was evaluated in porcine esophageal mucosa, which has been reported to possess a similar structure and lipid composition as that of human buccal mucosa [16]. The porcine esophagus was obtained from a local abattoir (Abattoir de Loëx Sàrl; Bernex, Switzerland) and transported to the laboratory in ice cold Krebs-Ringer bicarbonate buffer (KRB, pH 7.4). Esophagus was longitudinally dissected and rinsed with isotonic saline. The mucosa was separated from the underlying muscular layer with a scalpel. The full-thickness mucosa was cut into 2 cm^2^ circular pieces and immediately used for the transport studies.

#### 2.4.2. Iontophoretic Transport Study

The iontophoretic setup used for evaluation of mucosal iontophoresis was similar to that described in our earlier studies [5,25]. The mucosal tissue was clamped in vertical Franz diffusion cells (diffusion area 0.6 cm^2^). After equilibrating the mucosa for 30 min with PBS (pH 7.4), 0.5 mL of free-form drug solutions or drug-loaded iontosomes were placed in the donor compartment. The receiver compartment was filled with 4.5 mL of PBS containing 0.1% Tween 80. The positive electrode (anode) was connected to the donor compartment via a salt bridge assembly (3% agarose with 100 mM NaCl), while the receptor compartment contained the negative electrode (cathode). Constant current (0.5 mA/cm^2^) was applied using Ag/AgCl electrodes connected to a power supply (Kepco^®^ APH 1000 M; Flushing, NY, USA). After iontophoresis for either 10 or 20 min, a 1 mL aliquot was withdrawn from the receptor compartment to quantify the amount of drug permeated across the mucosa. Passive permeation experiments using a similar setup but without current application served as controls. The deposited amounts of DTX and CDDP were extracted by cutting the mucosa samples into small pieces and soaking in 10 mL of extraction media for 12 h. DTX was extracted using a 30:70 mixture of ammonium acetate (5 mM) and methanol; CDDP was extracted using PBS (pH 7.4) containing 1% (*w*/*v*) Triton X-100 at 45 °C. The extraction methods were validated by spiking mucosa samples with known amounts of drugs. The extracts were filtered using 0.22 µm PTFE filters (Simplepure-PTFE, BGB Analytik SA) and processed for HPLC-UV analysis as described in the Addendum.

#### 2.4.3. CLSM Microscopy

The mucosal transport of iontosomes was also visualized using confocal laser scanning microscopy to determine the penetration pathways for such nanocarrier systems in the oral mucosa. For this, the iontophoretic transport study was performed as described above but using the fluorescent iontosomes. At the end of the experiment, the mucosa was washed under running tap water and fixed using 4% PFA in PBS. Transverse sections (10–20 µm thick) of the PFA-fixed tissue were obtained using a cryomicrotome (Leica Microsystems GmbH; Nussloch, Germany). The sections were mounted on glass slides, counterstained for nuclei with Hoechst 33,258, and scanned using a Zeiss LSM700 microscope (Carl Zeiss Microscopy GmbH, Jena, Germany).

### 2.5. Statistical Analysis

Data were expressed as mean ± SD. Outliers were determined using the Grubbs test. Results were statistically evaluated using Student’s *t*-test. The level of significance was fixed at α = 0.05.

## 3. Results and Discussion

### 3.1. Preparation and Characterization of Iontosomes

Liposomes are versatile nanocarriers that enable the encapsulation of both lipophilic and hydrophilic drugs; the former are held within the lipid bilayer, while the latter are entrapped inside the aqueous core of the liposomes [26]. Here, we report the successful co-encapsulation of a hydrophilic–lipophilic drug combination (CDDP and DTX) in cationic liposomes. Initially, the liposomes were prepared by the conventional lipid film hydration method, wherein a DTX containing lipid film was hydrated with an aqueous solution of CDDP. However, the entrapment of CDDP in these liposomes was inadequate (6.3 ± 0.8%) (Table 1, Lip-1). The aqueous volume entrapped within such liposomes is generally less than 10% of the total volume; consequently, a large amount of CDDP remained unentrapped and outside the liposomes [27]. Hence, a modified method was employed to improve CDDP entrapment (Figure 2).

**Figure 3:**
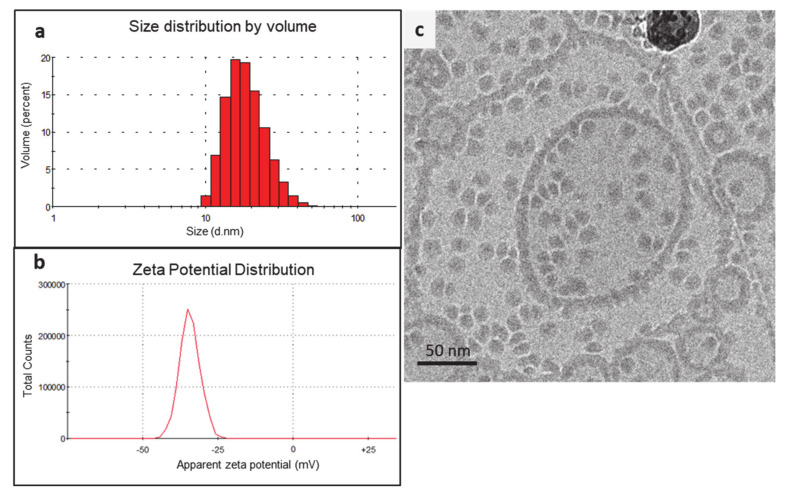
(**a**) Particle size distribution, (**b**) zeta potential distribution, and (**c**) cryo-TEM images of the formulated CDDP–DPPG reverse micelles.

In the modified method, CDDP was first formulated into reverse micelles using an anionic lipid, DPPG. The formation of reverse micelles was driven by the aquation of CDDP, which is a process involving hydrolysis of the chloride atoms in CDDP and their replacement by water molecules. The aquated CDDP carried two positive charges, and hence, it instantaneously formed reverse micelles with the DPPG in 30% ethanol [18]. The reverse micelles obtained displayed a Z-average size of 15.6 ± 5.2 nm and exhibited a negative zeta potential of −32.8 ± 8.6 mV (Figure 3a,b). The cryo-TEM images displayed uniformly distributed micelles with a similar size range (Figure 3c). The presence of an electron-dense core in the micelles further confirmed that the CDDP had been successfully loaded. The cryo-TEM micrographs also indicated that some of the reverse micelles aggregated into strings and larger particles, which explained the relatively broad size distribution observed during DLS measurements (Figure 3a). The rationale for using anionic CDDP–DPPG reverse micelles was that they could provide higher CDDP loading in the liposomes via electrostatic interaction with the cationic lipid film.

In the next step, the DTX-loaded cationic lipid film was hydrated with the CDDP–DPPG reverse micelles to obtain the iontosomes. A series of formulations were prepared by adding different volumes of the reverse micelle solutions (Table 1). As shown in Table 1, the formulated iontosomes displayed similar Z-average diameters with a positive zeta potential due to the presence of DOTAP, which has a positively charged head group. Increasing the volume of CDDP–DPPG reverse micelles added from 2.5 mL to 10 mL lowered the zeta potential of formulated iontosomes from 42.3 ± 4.6 mV to 8.6 ± 2.4 mV, indicating the neutralization of cationic charge on lipid bilayers by the anionic reverse micelles.

As expected, the addition of increasing volumes of reverse micelles increased the amount of CDDP entrapped in the iontosomes, as indicated by the ratio of CDDP:DTX content in the iontosomes (Table 1). The content ratio increased linearly from 0.7 in Lip-2 to 2.7 in Lip-4 when the volume of reverse micelles was increased from 2.5 to 10 mL. However, this adversely affected the content and encapsulation efficiency (EE) of DTX, especially in Lip-4. It is possible that some of the reverse micelles were also incorporated in the lipid bilayer of the iontosomes, displacing DTX from the lipid bilayer. Lip-3 with a CDDP:DTX content ratio of 1.28 was selected for further studies due to the positive zeta potential it offered with a relatively high EE for both drugs. In addition, the CDDP:DTX ratio was similar to the ratio of the clinical doses of these drugs when used in combination chemotherapy of head and neck cancers [28,29]. The combination regimen with a similar dose ratio (75–100 mg/kg CDDP with 75 mg/kg DTX) has been shown to offer higher tumor response and survival rates relative to the standard regimen of CDDP/5-FU in treatment of recurrent head and neck cancer. Lip-3, which was selected for further experiments, had a mean diameter of 109.8 ± 12.4 nm with a polydispersity index (PDI) of around 0.25 and displayed a positive zeta potential of 37.3 ± 1.9 mV. The CDDP and DTX contents in Lip-3 were 0.84 ± 0.05 mg/mL and 0.66 ± 0.03 mg/mL, respectively.

### 3.2. Cryo-TEM Analysis of the Co-Encapsulated Iontosomes

**Figure 4:**
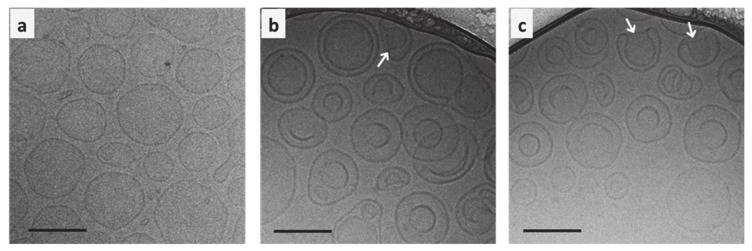
Cryo-TEM images of (**a**) conventional liposomes and (**b**,**c**) iontosomes co-encapsulating cisplatin and docetaxel. The white arrows indicate the interlamellar attachments, which is a defect commonly observed during the formation of bilamellar vesicles. The scale bars represent 100 nm.

The cryo-TEM micrographs clearly demonstrate the structural differences between conventional liposomes and iontosomes (Figure 4). The conventional DOTAP/Lipoid-S75 liposomes prepared without CDDP–DPPG reverse micelles were predominantly spherical and unilamellar (Figure 4a). On the other hand, a majority of the iontosomes had bilamellar structures (Figure 4b,c). This was expected, as the liposomes with highly flexible lipid bilayers are known to undergo vesicle deformation and bilayer invaginations. The otherwise rigid DOTAP/Lipoid-S75 bilayers were made flexible by the small amount of ethanol present in the CDDP–DPPG reverse micelles. Ethanol is known to cause membrane fusion and interdigitation in the phosphatidylcholine based bilayers, leading to the formation highly deformable liposomes (ethosomes) [30,31]. However, unlike the previously reported ethosomes, the formulated iontosomes were uniform in size, shape, and lamellarity. This can be explained by the electrostatic interactions between the cationic lipid bilayer and anionic reverse micelles in the iontosomes. It is possible that the anionic reverse micelles were sandwiched between multiple cationic vesicles during the hydration step. Then, the extrusion process caused the inversion of larger vesicles and resulted in complete invagination of the smaller vesicle with the sandwiched reverse micelles, leading to the formation of bilamellar vesicles. A similar mechanism has been reported for the DOTAP liposomes encapsulating DNA [32]. Some of the iontosomes also showed interlamellar attachments (indicated by white arrows) resulting in semi-toroidal structures. These structures are the intermediates formed during the process of membrane fusion and are commonly observed during the formation of bilamellar vesicles [33]. The mean size of all formulations was in the range 100–125 nm, which was in good agreement with the DLS data (Table 1).

### 3.3. Iontophoretic Stability and In Vitro Drug Release

**Figure 5:**
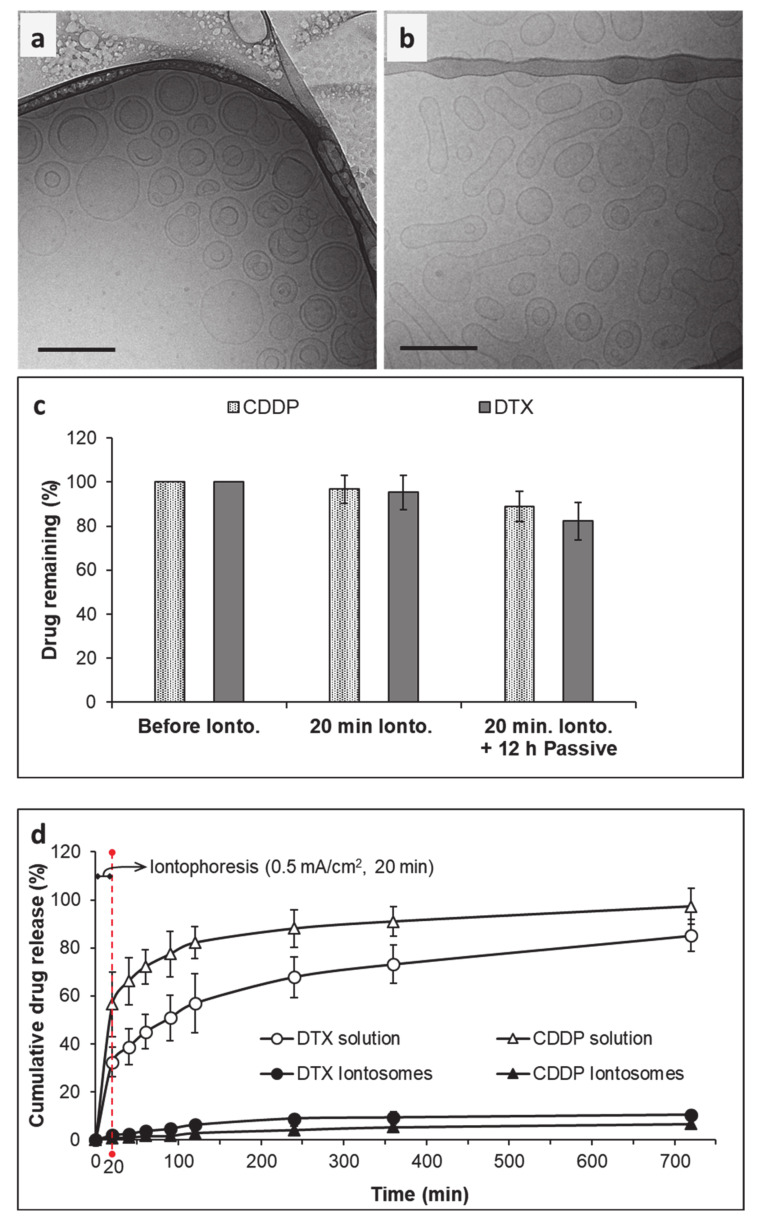
Effects of iontophoresis on the characteristics of iontosomes: the cryo-TEM images (**a**) before and (**b**) after iontophoresis demonstrate the impact of electric current application on iontosome morphology (scale bars represent 100 nm), (**c**) illustrates the percentage of drug content retained in the iontosomes before and after 20 min of iontophoresis followed by 12 h of passive treatment, (**d**) shows in vitro release behavior of the iontosomes before and after iontophoresis followed by 12 h of passive treatment; free-form drug solutions were used as controls.

The iontosomes containing co-encapsulated CDDP and DTX were examined for their ability to undergo shape deformation under the iontophoretic conditions (0.5 mA/cm^2^ for 20 min) to be used for the delivery studies. The cryo-TEM micrographs clearly showed the shape deformation of iontosomes after application of the iontophoretic current (Figure 5). The untreated iontosomes had a spherical bilamellar structure before being exposed to the electric current (Figure 5a). However, after iontophoresis, most of the iontosomes had an elongated shape (Figure 5b). It was postulated that this shape deformation was due to the differences in the charge distribution within the iontosomes. During anodal iontophoresis (as used in this case), the positively charged molecules electromigrate from the electrode compartment toward the adjoining membrane; simultaneously, the anionic species are attracted toward the anode [34]. Therefore, when the iontosomes are exposed to the electric current, the cationic lipids electromigrate toward the receiver compartment, whereas the anionic micelles are attracted to the anode. The opposing movements of components within the same vesicle would result in the elongation of the flexible iontosomes (Figure 1).

The cryo-TEM images indicated that the diameters of the elongated iontosomes were between 40 and 50 nm. However, the DLS results indicated that the mean particle sizes and PDI of the iontosomes increased slightly after the iontophoresis (Table 2). This disparity could be due to the elongated shapes of the iontosomes, since the hydrodynamic diameter reported by the DLS is influenced by the shape of particles. It is well-known that DLS measures the Brownian motion of the particles and relates it to their size [35]. The larger the particle, the slower the Brownian motion; however, this holds true only if the particles are spherical. The diffusion speeds of the rod-shaped particle are slower, and hence, the hydrodynamic sizes calculated by DLS were higher.

Next, the ability of iontosomes to retain the loaded drugs under the iontophoretic conditions was evaluated (Figure 5c). The amounts of CDDP and DTX remaining in the formulation after iontophoresis (0.5 mA/cm^2^ for 20 min) were similar to those observed in the untreated iontosomes (96.83 ± 6.3 and 95.27 ± 7.8 for CDDP and DTX, respectively). DOTAP is known to improve the physical stability of phosphatidylcholine-based liposomes by increasing lipid bilayer fluidity, thereby efficiently retaining bulky hydrophobic drugs such as DTX in the lipid domains of the liposomes [36]. Similarly, the electrostatic attraction between the cationic lipid bilayer and the anionic CDDP–DPPG reverse micelles helped to retain CDDP inside the liposomes during iontophoresis for 20 min. Furthermore, only small amounts of DTX and CDDP were released from the formulations during the subsequent in vitro release study.

After the iontophoretic treatment, the in vitro release behavior of the iontosomes was evaluated for 12 h. The CDDP and DTX solutions were used as controls to confirm that the experimental set-up did not hinder the diffusion of drugs across the selected dialysis membrane. As expected, the solution formulations of both CDDP and DTX easily diffused across the dialysis membrane into the receiver chamber of Franz diffusion cells, releasing over 50% of the drug content within 2 h. In contrast, sustained release was observed from the co-encapsulated iontosomes. The cumulative amounts of CDDP and DTX released into the receiver chambers after 12 h were 6.7 ± 1.7% and 10.4 ± 1.9%, respectively. These findings clearly demonstrated the potential of the iontosomes as carriers to enable the controlled and sustained release of chemotherapeutics into cancerous tissue.

### 3.4. Evaluation of Cytotoxic Activity In Vitro

**Figure 6:**
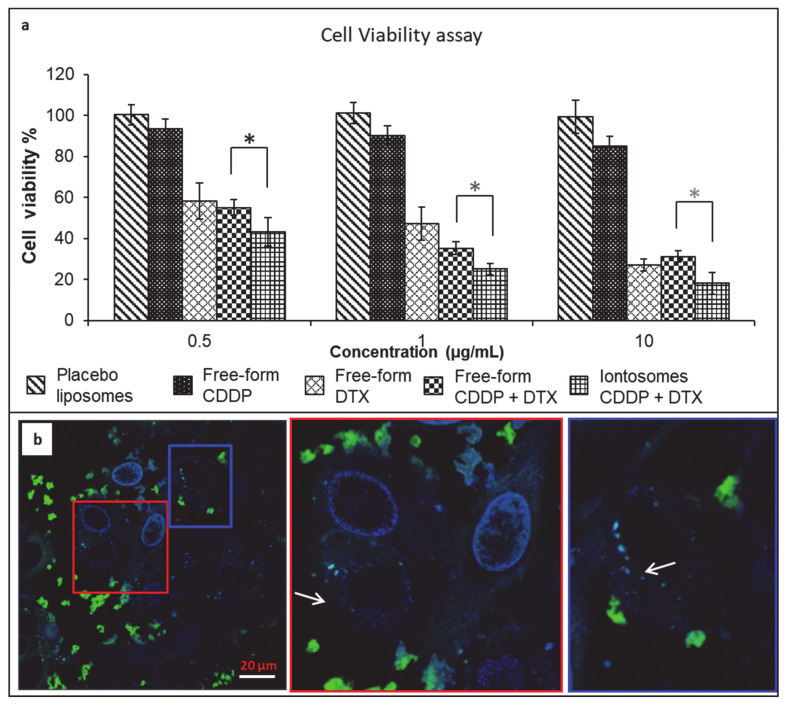
(**a**) Viability of HeLa cells incubated with placebo iontosomes, free-form cisplatin (CDDP), free-form docetaxel (DTX), their combinations in solution formulation or co-encapsulated iontosomes. The CDDP and DTX concentrations used in solution formulations were similar to those observed in the iontosomes; the DTX concentration ranged from 0.01 to 10 μg/mL, the corresponding CDDP concentrations were 0.013 to 12.8 μg/mL. Cell viability was determined by the MTT assay. Untreated cells were used as controls. The results are expressed as mean ± standard deviation of five measurements. (**b**) Confocal images showing the adhesion and uptake of fluorescent iontosomes by HeLa cells. The white arrows in the magnified views show the transport of iontosomes to the cell nuclei. (*, *p* < 0.05).

In order to verify that the DTX and CDDP co-encapsulated in the iontosomes retained their bioactivity, cytotoxicity assays were performed in vitro using HeLa cells (Figure 6). The solution formulations of DTX and CDDP were used as the reference. Before incubation with HeLa cells, the formulations were sterilized by aseptic filtration to exclude the possibility of any contamination. The formulations were diluted to achieve predetermined DTX and CDDP concentrations between 0.5 and 10 µg/mL. Figure 6a shows the effect of the co-encapsulated iontosomes, DTX solution and CDDP solution at equivalent drug concentrations on cell viability in vitro. The percentage of viable cells was quantified using an MTT assay. The results indicated that the blank iontosomes were non-toxic to the cells, as no significant cytotoxicity was observed even at the highest iontosome concentration. The HeLa cells appeared less sensitive to CDDP in comparison to DTX, since even at the highest CDDP concentration tested (34.4 nmol/mL), over 85% of cells were still viable. On the other hand, formulations containing DTX exhibited a dose-dependent cytotoxicity on HeLa cells. In addition, the drug-loaded iontosomes exhibited a slightly better cytotoxicity than the free-form DTX or its combination with the CDDP.

Figure 6b shows confocal images that demonstrate the cellular uptake of fluorescent iontosomes. The iontosomes were successfully internalized by the HeLa cells and were transported to the nuclei (white arrows). Some of the iontosomes also appeared to adhere to the cell surfaces due to their cationic zeta potential. These results clearly indicated that the formulated iontosomes retained the chemotherapeutic potential of DTX and CDDP.

### 3.5. Buccal Delivery of Iontosomes

Porcine buccal mucosa is generally considered to be a good model to predict permeation in human oral mucosa [5,16]. However, limited availability of this tissue prompted the use of porcine esophageal mucosa, which has been shown to possess similar structure, composition, and barrier properties to the buccal tissue [37]. Furthermore, the permeabilities of small molecule drugs such as carbamazepine and fentanyl citrate across the two epithelial barriers were reported to be comparable [16,38]. Hence, the iontophoretic buccal delivery of the CDDP and DTX formulated in iontosomes was tested using porcine esophageal mucosa and solution formulations of CDDP and DTX containing equivalent concentrations were used as controls.

The passive delivery of CDDP and DTX solutions or co-encapsulated iontosomes for 10 or 20 min resulted in concentrations in the receiver compartment that were below the limit of quantification. Furthermore, the results indicated that only the CDDP in solution form permeated (0.92 ± 0.13 µg/cm^2^) across the mucosal tissue following iontophoresis (0.5 mA/cm^2^, 20 min).

**Figure 7:**
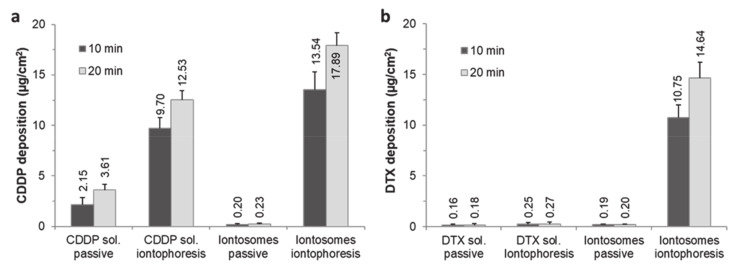
Mucosal deposition of (**a**) CDDP and (**b**) DTX following passive diffusion or co-iontophoresis (at 0.5 mA/cm^2^) for either 10 or 20 min using their aqueous solutions (at pH 7.0) or co-encapsulated iontosomes. The CDDP and DTX concentrations were 0.84 and 0.66 mg/mL, respectively. The results are expressed as mean ± standard deviation of four measurements.

The amounts of each drug deposited in the mucosa are shown in Figure 7. Passive application of the CDDP solution for 10 or 20 min resulted in mucosal deposition of 2.15 ± 0.69 and 3.61 ± 0.55 µg/cm^2^, respectively (Figure 7a). Iontophoresis resulted in CDDP deposition of 9.70 ± 1.07 and 12.53 ± 0.92 µg/cm^2^ after current application for 10 and 20 min, respectively—corresponding to 4.5- and 3.4-fold increases over the passive controls. In contrast to the CDDP solution, both passive and iontophoretic delivery of DTX solution for 10 and 20 min resulted in almost negligible deposition of DTX in the mucosa (passive—0.16 ± 0.11 and 0.18 ± 0.12 µg/cm^2^ and iontophoresis—0.25 ± 0.16 and 0.27 ± 0.16 µg/cm^2^) (Figure 7b). CDDP is positively charged under physiologic conditions due to the aquation process, which favors its anodal iontophoretic delivery. DTX being a larger molecule does not penetrate the mucosa easily by passive diffusion in such short time periods.

The deposition of CDDP and DTX from the iontosomes was also compared following passive and iontophoretic delivery for 10 and 20 min: the mucosal deposition of both drugs was significantly greater after iontophoresis. Iontophoretic delivery for 10 and 20 min led to 67.7- and 77.8-fold increases in the buccal deposition CDDP (passive—0.20 ± 0.07 and 0.23 ± 0.10 µg/cm^2^ and iontophoresis—13.54 ± 1.78 and 17.89 ± 1.28 µg/cm^2^ for 10 and 20 min applications, respectively). For DTX, similar increases in deposition of 56.6- and 73.2-fold, respectively, were observed following iontophoresis for 10 and 20 min (passive—0.19 ± 0.09 and 0.20 ± 0.05 µg/cm^2^ and iontophoresis—10.75 ± 1.25 and 14.64 ± 1.54 µg/cm^2^ for 10 and 20 min applications, respectively).

Interestingly, the CDDP:DTX deposition ratios (1.26 and 1.22) after iontophoretic delivery of iontosomes were similar to their initial content ratio (1.28), suggesting that the deposited CDDP and DTX were still associated with the iontosomes and that the iontosomes remained intact during transport. The disintegration of iontosomes and/or release of loaded drugs during the iontophoretic transport would have led to a reduced deposition of DTX or improved deposition of CDDP, as seen during the iontophoretic transport of their solution formulations. To demonstrate the therapeutic relevance of local iontosomal delivery in oral cancers, the CDDP and DTX amounts deposited in the mucosa samples were converted into the approximate tissue-level concentrations (Table 3). These levels were found to be higher by multiple orders of magnitude than the reported IC_50_ values for CDDP (670 nM) and DTX (70 nM) in TE-2 cells, which is a human esophageal squamous cell carcinoma cell line. Unfortunately, it was not possible to make a similar extrapolation for intratumoral levels of CDDP and DTX; to our knowledge, there are no available data on the intratumoral levels of these drugs.

### 3.6. Visualization of Mucosal Transport of Iontosomes

**Figure 8:**
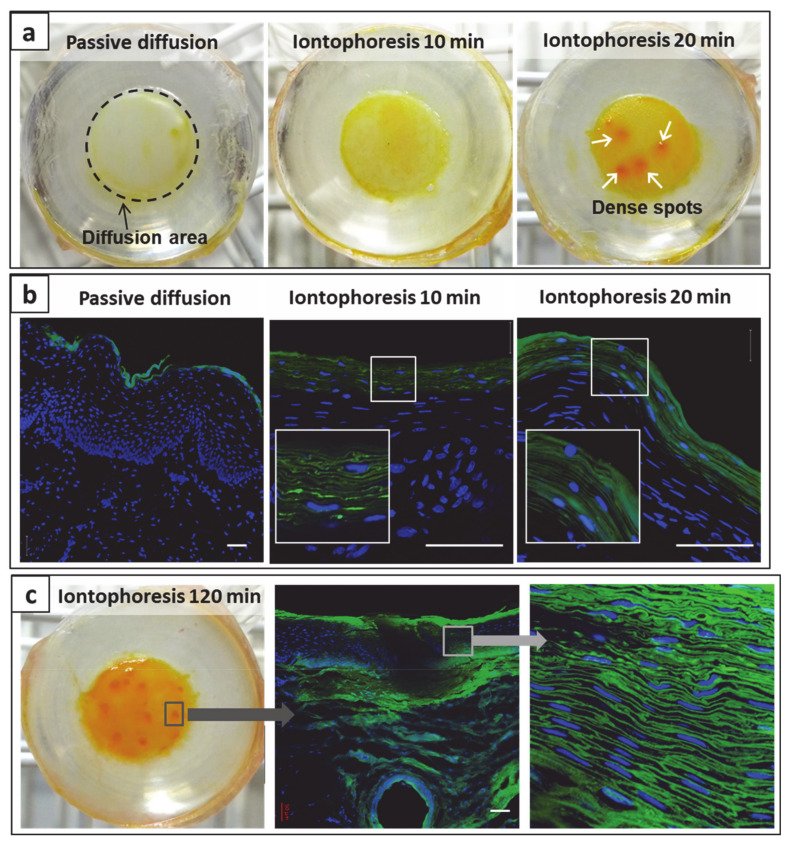
Deposition and transport of iontosomes through mucosal tissue after passive or iontophoretic delivery for different durations. (**a**) shows the photographs of mucosal surface exposed to iontosomes via passive (20 min) or iontophoretic delivery (10 and 20 min). Note that the dense spots (white arrows) formed due to the accumulation of iontosomes after iontophoresis for 20 min. (**b**) shows the corresponding confocal images of the same mucosal tissues. The insets show the magnified views of the smaller white squares in the respective images (scale bar represents 100 μm). (**c**) shows the mucosal transport of iontosomes after iontophoresis for 120 min. A transverse section through the dense spot (confocal images) shows that the iontosomes can penetrate into the deeper layers of mucosa through intercellular spaces. (scale bar represents 50 μm).

The incorporation of fluorescent Lipoid-S75 in iontosomes allowed their visualization during iontophoretic transport across the mucosal tissue using CLSM. Visual inspection of mucosa treated with the fluorescent iontosomes for different durations (Figure 8a) show that at a macroscopic level, iontophoresis led to a yellowish staining of mucosal surfaces with fluorescent iontosomes as compared to passive delivery. This was more pronounced, with an increased duration of iontophoresis indicating that more iontosomes were adhering to mucosal surfaces (Figure 8a–middle and right images showing iontophoresis for 10 and 20 min, respectively). The iontosomes also appeared to accumulate at certain dense spots on the mucosa following iontophoresis for 20 min (Figure 8a, white arrows). These spots were possibly low-resistance regions in the mucosa that allowed an enhanced passage of charged species during iontophoresis. In the case of transdermal iontophoresis, the charged species can follow an appendageal pathway through the sweat glands and/or pilosebaceous units as the diffusional resistance of the skin is lower in such regions [39]. However, experiments with cell culture-based living skin equivalents have suggested that these appendages are not essential for iontophoresis to be successful [40]. Furthermore, it was also suggested that transient pores may be created through lipid reorganization by the applied electric field [41,42]. The transdermal iontophoretic transport of several compounds has been shown to occur through the intercellular route; it was claimed that the application of electrical current makes the stratum corneum lipid lamellae more accessible to water and ions. We hypothesized that the iontosomes also enter the epithelium through intercellular pathways owing to their flexible structures. To confirm this, transverse sections of the mucosa samples were observed using CLSM (Figure 8b).

After passive treatment, most of the iontosomes were present on the mucosal surface. In contrast, iontophoresis led to a visually significant improvement in mucosal penetration of the iontosomes. Magnified sections of the confocal images clearly indicated that the major pathway for penetration of iontosomes was through the intercellular spaces. As discussed earlier, these intercellular spaces are known to be narrower than 20 nm; hence, without iontophoresis, the iontosomes remain on the surface of the mucosa. However, during iontophoresis, the iontosomes undergo shape deformation and are carried into the mucosa due to their cationic surface charge. The penetration depths appeared similar for either 10 or 20 min iontophoresis (≈40–50 µm). This was surprising, so an additional study was performed with a longer duration of iontophoresis (120 min) (Figure 8c). The macroscopic examination indicated that the number of dense spots was increased. A similar activation of low-resistance pores has been shown to occur during transdermal iontophoresis in hairless mice; it was shown that the spatial density of current-carrying pores increased from 0 to 100–600 pores/cm^2^ during the first 30–60 min of iontophoresis [43]. Our findings suggested that the buccal mucosa behaved similarly to skin and that iontophoresis created low-resistance pores that resulted in the accumulation of iontosomes as discrete spots on the epithelial surfaces. The confocal images of transverse sections through these spots displayed the penetration of iontosomes through the epithelium and down into the submucosa. Overall, the results indicated that the penetration depth of the iontosomes can be controlled by varying the duration of iontophoresis.

## 4. Conclusions

The results presented here demonstrate that two physicochemically incompatible chemotherapeutic agents, CDDP and DTX, could be encapsulated in electroresponsive shape-deformable iontosomes and, furthermore, that this could be achieved at high entrapment efficiencies. The iontosomes carried a cationic surface charge with flexible lipid bilayers; however, these properties were not sufficient for their spontaneous penetration into the mucosa. The application of iontophoresis led to shape deformation of the iontosomes and significantly enhanced their mucosal penetration through the intercellular spaces between epithelial cells—visualization of the penetration pathway was achieved using CLSM. Quantification of the amounts of CDDP and DTX present in the mucosa after passive and iontophoretic administration showed that already after 10 min, there were 67.7- and 56.6-fold increases in mucosal deposition upon current application. The ratio of the amounts of the two drugs present in the mucosa was similar to that found in the iontosomes, suggesting that the deposited CDDP and DTX had been delivered through the electrotransport of intact iontosomes into the membrane. The combination of iontosomes with electrically-assisted delivery appears to be a promising method for delivering multiple chemotherapeutics selectively to oral mucosa and offers a non-invasive targeted approach for the treatment of cancers that also limits their systemic absorption and the associated risk of off-site side effects.

## Figures and Tables

**Table 1:** Composition, mean diameter, polydispersity index (PDI), zeta potential, loading efficiency, and drug content of formulated iontosomes.

Formulation	Vol. R.M. (mL)	Diameter (nm)	PDI	Zeta Pot. (mV)	EE (%)		Drug Content (mg/mL)		Content Ratio
					CDDP	DTX	CDDP	DTX	
Lip-1	0.0	120.3 ± 13.9	0.29	52.8 ± 2.1	6.3 ± 0.8	87.3 ± 4.1	0.06 ± 0.01	0.71 ± 0.03	0.1 ± 0.0
Lip-2	2.5	107.5 ± 15.5	0.21	42.3 ± 4.6	91.1 ± 3.1	84.2 ± 4.9	0.46 ± 0.02	0.68 ± 0.04	0.7 ± 0.0
Lip-3	5.0	109.8 ± 12.4	0.25	34.3 ± 3.6	84.4 ± 4.9	81.7 ± 3.7	0.84 ± 0.05	0.66 ± 0.03	1.3 ± 0.1
Lip-4	10.0	120.4 ± 17.1	0.35	8.6 ± 2.4	51.8 ± 6.5	48.8 ± 8.2	1.04 ± 0.13	0.39 ± 0.07	2.7 ± 0.8

Note: Vol. RM: volume of reverse micelles used, PDI: polydispersity index; EE: encapsulation efficiency; CDDP: cisplatin, DTX: docetaxel, Content ratio: the ratio of CDDP:DTX amounts present in final formulation. Lip-1 comprises conventional liposomes prepared using CDDP solution.

**Table 2:** Effect of iontophoresis on the characteristics of the iontosomes.

Condition	Iontosome Size (nm)	PDI	Zeta Pot. (mV)
Before iontophoresis	109.8 ± 12.4	0.25	42.3 ± 4.6
Iontophoresis:10 min	125.8 ± 24.5	0.29	40.3 ± 7.2
Iontophoresis:20 min	139.8 ± 27.4	0.37	41.7 ± 5.5

**Table 3:** Therapeutic relevance of the CDDP and DTX amounts deposited in the mucosa.

Drug	Iontophoresis Duration	Deposition (µg/cm^2^)	Approx. Mucosal Conc. (µM) *	X-Fold Higher Than IC_50_
CDDP	10 min	13.5 ± 1.8	835.8 ± 109.7	1247.45
	20 min	17.9 ± 1.3	1104.6 ± 079.1	1648.62
DTX	10 min	10.8 ± 1.3	246.5 ± 028.7	3521.10
	20 min	14.6 ± 1.5	335.6 ± 035.3	4794.55

* These amounts were calculated by considering that the mucosa samples had an average thickness of ≈0.9 mm, i.e., 0.09 cm, and that the area was 0.6 cm^2^, meaning the mucosal volume was 0.054 cm^3^.

## Data Availability

The data presented in this study are available on request from the corresponding author.

## Supplementary Materials

The following are available online at https://www.mdpi.com/1999-4923/13/1/88/s1: Figure S1: Chromatograms of mucosa, extraction solvent and DTX standard (10 µg/mL); Figure S2: Formation of the derivative of CDDP with DDTC to form CDDP-DDTC; Figure S3: Chromatograms of mucosa, DDTC alone, complex CDDP-DDTC standard (12.5 µg/mL); Table S1: Intra- and inter-day accuracy and precision values for DTX quantification method; Table S2: Intra- and inter-day accuracy and precision values for CDDP-DDTC quantification method.
