# Supplementary material for: Iontosomes: Electroresponsive Liposomes for Topical Iontophoretic Delivery of Chemotherapeutics to the Buccal Mucosa
Source: Pharmaceutics. 2021 Jan 11;13(1):88. doi: 10.3390/pharmaceutics13010088 (PMC7826915; doi:10.3390/pharmaceutics13010088)
Supplement: Supplementary file 1 [file pharmaceutics-13-00088-s001.pdf]

pharmaceutics

Supplementary Materials

# Iontosomes: Electroresponsive Liposomes for Topical Iontophoretic Delivery of Chemotherapeutics to the Buccal Mucosa

**Kiran Sonaje, Vasundhara Tyagi, Yong Chen and Yogeshvar N. Kalia***

## 1. Validation of HPLC-UV method for quantification of DTX in mucosa samples

### *1.1. Specificity*

The method was specific for DTX quantitative analysis at 227 nm. DTX was eluted at 9.3 ± 0.3 min. Figure S1 presents the chromatogram for mucosa matrix, DTX extraction solvent (70:30 mix of methanol and ammonium acetate) and DTX 10 µg/mL standard. DTX stock solution (1 mg/mL) was prepared in methanol and further diluted in the extraction solvent to prepare working solutions. The volume of injection was 50 µL.

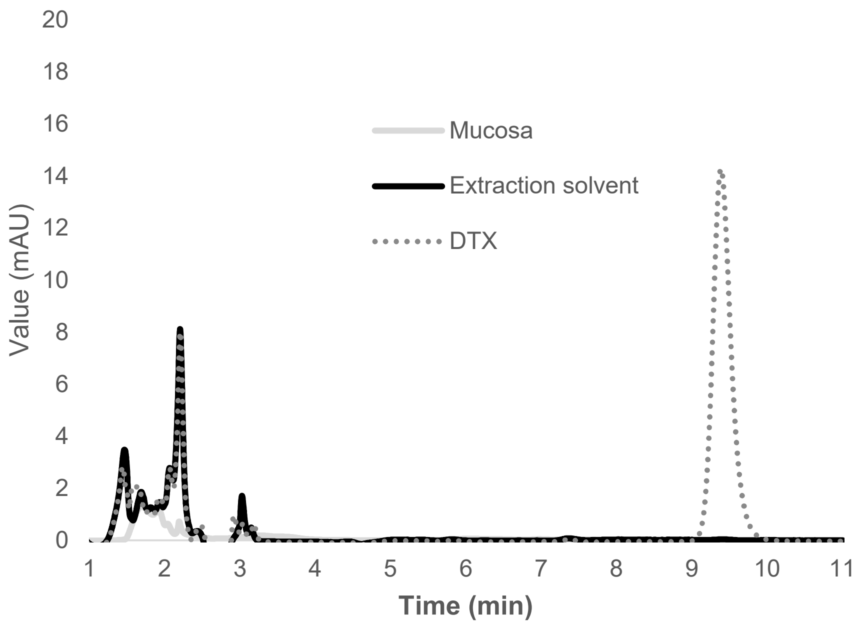


**Figure S1.** Chromatograms of mucosa, extraction solvent and DTX standard (10 µg/mL).

### *1.2. Limit of detection and limit of quantification*

The limit of detection (LOD) and limit of quantification (LOQ) were 0.05 µg/mL and 0.2 µg/mL, respectively.

### *1.3. Linearity*

The method was linear in the concentration range of 0.2 – 20 µg/mL with a $R^2$ of 0.99.

### *1.4. Accuracy and precision*

Intra- and inter-day accuracy and precision were calculated using 0.5, 1 and 10 µg/mL standard solutions. Table S1 shows the intra- and inter-day accuracy and precision values for DTX quantification method.

**Table S1. Intra- and inter-day accuracy and precision values for DTX quantification method**.

| | Intra-day | | | Inter-day 1 | | | Inter-day 2 | | |
|---|---|---|---|---|---|---|---|---|---|
| $[DTX]_{theo}$ (µg/mL) | $[DTX]_{mean}$ (µg/mL) | RSD (%) | Recovery (%) | $[DTX]_{mean}$ (µg/mL) | RSD (%) | Recovery (%) | $[DTX]_{mean}$ (µg/mL) | RSD (%) | Recovery (%) |
| 0.5 | 0.48 ± 0.00 | 1.18 | 96.79 | 0.46 ± 0.00 | 2.4 | 93.42 | 0.51 ± 0.01 | 2.34 | 98.64 |
| 1 | 1.02 ± 0.10 | 0.66 | 102.4 | 0.97 ± 0.01 | 1.15 | 97.27 | 0.92 ± 0.01 | 1.07 | 92.8 |
| 10 | 9.95 ± 0.06 | 1.18 | 99.51 | 10.0 ± 0.04 | 0.53 | 100.17 | 10.1 ± 0.21 | 2.08 | 101.2 |

The method was accurate and precise according to the validation guidelines [1].

## 2. Validation of HPLC-UV method for the quantification of CDDP in mucosa samples

### *2.1. Derivatization of CDDP*

Due to the low molar absorptivity of CDDP alone, indirect measurement of CDDP was performed via derivatization using diethyldithiocarbamate (DDTC). Aqueous CDDP readily reacts with the sulphur groups in DDTC to form the derivative DDTC-CDDP (Figure S2)

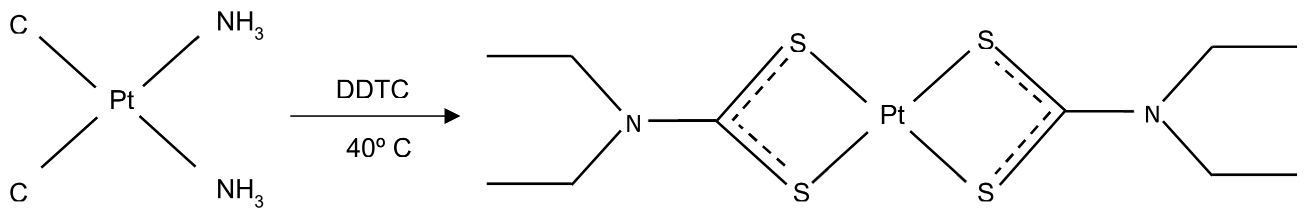


**Figure S2.** Formation of the derivative of CDDP with DDTC to form CDDP-DDTC.

Briefly, the extracted samples were centrifuged at 10,000 rpm for 15 minutes to separate the mucosa matrix. The supernatant was collected. After the addition of appropriate amount of 1% w/v DDTC in 0.1M NaOH, the mix was incubated at 40 degrees for 30 mins. The mix was then centrifuged at 10,000 rpm for 15 minutes. The supernatant was separated and reconstituted with acetonitrile. The sample was ready to be injected in HPLC-UV.

### *2.2. Specificity*

The method was specific for CDDP-DDTC quantitative analysis at 254 nm. CDDP-DDTC was eluted at 7.0 ± 0.2 min. Figure S3 presents the chromatogram for mucosa matrix, CDDP-DDTC 12.5 µg/mL standard and only DDTC. CDDP-DDTC stock solution (1 mg/mL) was prepared in dimethylsulfoxide (DMSO) and further diluted acetonitrile to prepare working solutions. The volume of injection was 50 µL.

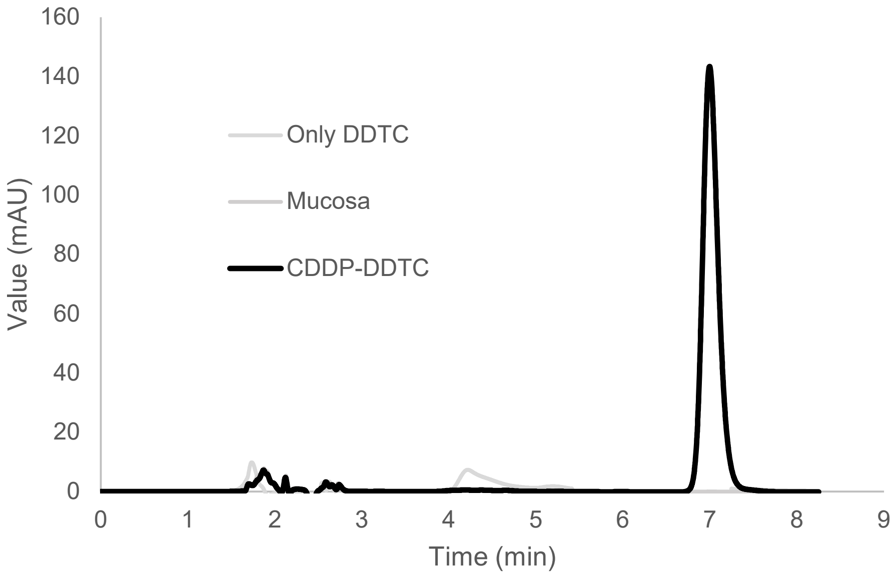


**Figure S3.** Chromatograms of mucosa, DDTC alone, complex CDDP-DDTC standard (12.5 μg/mL).

*2.3. Limit of detection and limit of quantification*

The limit of detection (LOD) and limit of quantification (LOQ) were 0.05 μg/mL and 0.3 μg/mL.

*2.4. Linearity*

The method was linear in the concentration range of 0.3 – 25 μg/mL with a $R^2$ of 1.

*2.5. Accuracy and precision*

Intra- and inter-day accuracy and precision were calculated using 1.5, 6.25 and 12.5 μg/mL standard solutions. Table S2 shows the intra- and inter-day accuracy and precision values for CDDP-DDTC quantification method.

**Table S2. Intra- and inter-day accuracy and precision values for CDDP-DDTC quantification method.**

| | **Intra-day** | | | **Inter-day 1** | | | Inter-day 2 | | |
|---|---|---|---|---|---|---|---|---|---|
| **[CDDP-DDTC]$_{theo}$ (μg/mL)** | **[CDDP-DDTC]$_{mean}$ (μg/mL)** | **RSD (%)** | **Recovery (%)** | **[CDDP-DDTC]$_{mean}$ (μg/mL)** | **RSD (%)** | **Recovery (%)** | **[CDDP-DDTC]$_{mean}$ (μg/mL)** | **RSD (%)** | **Recovery (%)** |
| 1.5 | 1.48 ± 0.01 | 0.58 | 95.46 | 1.43 ± 0.00 | 0.17 | 91.95 | 1.41 ± 0.01 | 0.72 | 90.74 |
| 6.25 | 6.30 ± 0.08 | 1.21 | 100.76 | 6.30 ± 0.00 | 0.1 | 101.04 | 6.45 ± 0.00 | 0.12 | 103.44 |
| 12.5 | 12.5 ± 0.21 | 1.28 | 100.61 | 12.9 ± 0.01 | 0.15 | 103.66 | 12.4 ± 0.05 | 0.42 | 99.94 |

The method was accurate and precise according to the validation guidelines.[1]

## Reference

1 International Conference on Harmonisation, Validation of Analytical Procedures: Text and Methodology Topic Q 2 (R1), 2005. Available online: https://www.gmp-compliance.org/guidelines/gmp-guideline/ich-q2r1-validation-of-analytical-procedures-text-and-methodology (accessed on 2 November 2020).
